# The role of endothelial-derived factors in neural tube development: implications for organoid models

**DOI:** 10.1098/rsob.240341

**Published:** 2025-06-11

**Authors:** Amy van der Hoven, Mubeen Goolam

**Affiliations:** ^1^ Department of Human Biology, University of Cape Town, Cape Town, South Africa; ^2^ Neuroscience Institute, University of Cape Town, Cape Town, South Africa

**Keywords:** neural tube, embryology, neural tube organoid, brain organoid

## Abstract

The study of the neural tube (NT), the antecedent structure of the central nervous system, is challenging due to the inaccessibility of the embryo. Thus, our understanding of this crucial timepoint in development is limited. The use of organoid models in recent years has proven immensely beneficial in the field of embryology, allowing the direct study of *in vitro* models of early neural development. As organoids advance in complexity, the vascularization of brain organoids has become a point of interest due to its significant role in neural development. This raises the question of whether the vascularization of NT organoids is necessary to improve their accuracy. This review summarizes the role of vascularization both during and before NT formation and explores the effects of endothelial-derived factors on this process. While the data indicate that vascularization is essential for proper NT formation, this review also highlights a significant gap in our knowledge and the need to clarify these interactions in order to generate more accurate organoid models.

**Figure:**
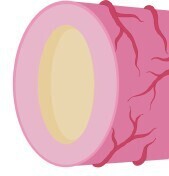

## 1. Introduction

The neural tube (NT) is a transient structure, emerging during embryonic development, which precedes the formation of the central nervous system (CNS) [1]. Understanding of the complex processes involved in mammalian NT formation has been hindered by implantation, which shields the embryo from direct observation [2]. For this reason, various models have been employed in an attempt to better elucidate mammalian NT formation [3] and the majority of our knowledge surrounding NT development is due to the use of animal models such as mice [4–7]. While this has allowed significant advancements in our understanding of embryogenesis, these animal models are unable to accurately recapitulate human development.

The recent development of organoid systems has presented as a novel approach to understand previously enigmatic aspects of embryonic development. Organoids are 3D aggregates of pluripotent stem cells that can be guided to differentiate into a tissue or organ of interest [8,9]. In this way, these *in vitro* stem cell models are able to accurately recapitulate the structure and function of a tissue or organ of interest without the need for embryos or animal models [10]. NT organoids (NTOs) are those that are directed to develop into the early NT, enabling direct study of both its formation and the disruption thereof which leads to congenital NT defects (NTDs) [3].

Despite the significant advancements in organoid technology over the past few years, a particularly intriguing limitation of current NTOs is their avascular nature [11]. Owing to the importance of the vascular system in development, the broader body of research dedicated to neural organoids has recently explored the vascularization of these models in order to more accurately demonstrate neural development and function. These vascular models have displayed higher levels of complexity and accuracy compared to their *in vivo* counterparts [12], revealing significant drawbacks of avascular models.

As organoid models, and the upper limit of their complexity, gain interest, it is of particular interest to researchers whether vascularization of NTOs is necessary to produce an accurate *in vitro* model of the NT. Considering our current knowledge of NT development relies heavily on these models, the lack of available vascularized NTOs raises the question, are our current modes of study accurate enough to make inferences about *in vivo* developmental processes? To explore this open question, this review will outline the known effects of vasculature on early CNS development, discuss research findings suggesting more enigmatic interactions between early vasculature and the NT, and consider potential improvements vascularization might afford NTOs based on current neural organoid models as well as current methods by which this might be achieved.

## 2. The role of endothelial-derived factors in neural tube development

### 2.1. Vascularization of the neural tube

The first indication of NT development is the emergence of the neural plate, a sheet of neuroepithelial cells derived from the ectodermal germ layer. As gestation progresses, this plate ingresses along the midline, forming two emerging neural folds that elevate on either side of the midline, eventually fusing to form a continuous tube [13].

**Figure 1.**
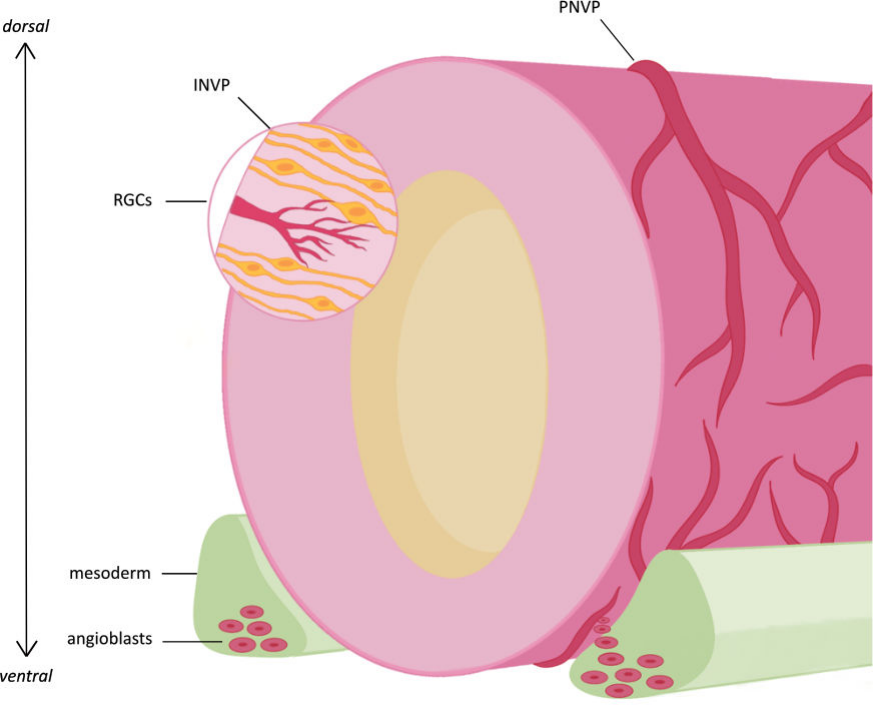
Vascularization of the neural tube. Following NTC, around week 6 in humans or E8.5 in mice, angioblasts are recruited from the surrounding mesoderm to form the PNVP, which surrounds the NT. The PNVP then sprouts into the NT, forming the INVP. Concurrently, neuroepithelial cells divide asymmetrically to form RGCs; the integrating INVP then forms associations with these RGCs.

NT formation, collectively termed neurulation, takes place roughly between embryonic day 7 (E7) and embryonic day 9.5 (E9.5) in mice and equivalently from gestational weeks 3–4 in humans [14]. This process is depicted in figure 1. While vascularization of the NT is not described before the onset of neurogenesis, these two processes are intimately linked. In fact, NT vascularization is initiated by the neural-derived pro-angiogenic factor vascular endothelial growth factor A (VEGF-A) to facilitate neural expansion shortly following the onset of neurogenesis [15,16].

In mice, this vascularization of the CNS commences between E7.5 and E9.5 [15], while this process occurs from roughly the sixth week of gestation in humans [17]. Following NT closure (NTC), endothelial progenitors termed angioblasts are recruited from the pre-somitic mesoderm to surround the NT in a primitive vascular mesh termed the perineural vascular plexus (PNVP) [15,18,19]. These vessels later sprout radially into the NT itself, developing the intraneural vascular plexus (INVP) [17]. In mice, PNVP establishment occurs between E8.5 and E10, with the INVP emerging at around E10.5 [20,21], while in humans, this is around week 8 [17].

Concurrently with INVP formation, neuroepithelial cells switch from symmetric divisions and the expansion of NSC pools to asymmetric divisions producing radial glial cells (RGC) [17,22]. These RGCs extend their processes along the apicobasal axis of the NT and angiogenic sprouts of the INVP associate with and align themselves with RGC fibres, laterally branching out to anastomose in the ventricular zone enabling the formation of the subventricular vascular plexus (SVP) [23–25].

### 2.2. The role of vasculature and endothelial-derived factors in neural development

Perhaps the most direct and predictable mechanism by which vascularization impacts neural development is the efficient transport of oxygen and nutrients alongside the removal of metabolites. This function is vital throughout mammalian tissue but more profoundly in highly proliferative regions [26]. Initially, the function of blood vessels was described solely in this manner; however, more recent findings have revealed that blood vessels also contribute to CNS development. While the reliance on signalling cues from neural cells on developing vasculature has been extensively reported [23,27,28], in a reciprocal manner, endothelial cells (ECs) are now understood to play an instructive role in the development of the early CNS. Thus, an important aspect of embryogenesis is the effective crosstalk between these developing systems.

Although the mechanisms by which ECs interact with developing neural cells are still not entirely understood [28], it has been suggested that ECs are involved in the regulation of neural proliferation, migration, and differentiation, commanding an important role in neurogenesis and neural progenitor cell (NPC) fate determination [23].

The precise balance of neuronal and glial generation is essential for proper nervous system development [29,30]. Furthermore, it has been suggested that dysregulation of neural stem cell proliferation can lead to neurodevelopmental disorders as well as defects in NT formation [31]. As a result, tight regulation of this process is critical. Both *in vitro* [32] and *in vivo* [33] mouse studies have reported that embryonic endothelial-derived soluble regulatory signals are able to support NPC expansion and delay neurogenesis. ECs have also been shown to provide secreted factors that serve to stimulate the expansion of neurospheres, neural stem cells isolated and expanded *in vitro* [34], significantly more than in neurospheres lacking ECs [32]. It has also been suggested that expansion and/or differentiation of neural progenitor subclasses is spatiotemporally associated with blood vessels, suggesting that vascularization plays a significant role in the expansion of progenitor subclasses [35].

Not simply to meet metabolic needs in proliferating cells, the oxygen supply in the developing CNS enables NPC fate commitment and neurogenesis via oxygen-sensitive signalling pathways. The role of oxygen-sensitive signalling is especially evident in hypoxia-induced factor 1α (HIF1-α), which plays a vital role in NPC fate commitment.

The neurogenic niches are initially hypoxic, a conditional requirement for NSC proliferation. During early cortical development, RGCs located in the ventricular zone switch from cell expansion to differentiation to generate neural progenitors that will later give rise to neurons and glia. The relief of hypoxia by angiogenesis in the neurogenic niche correlates spatiotemporally with RGC differentiation into progenitor populations. In this way, oxygenation directs progenitor fate. This mechanism is regulated by the NSC target, HIF1-α, where HIF1-α is activated in hypoxic conditions leading to increased NSC expansion while restraining differentiation of progenitor cells and neurogenesis [36]. Conversely, oxygenation destabilizes HIF1-α and induces increased NSC differentiation while limiting expansion [37]. In this way, vascularization appears to play a role in the patterning of the NT by secreting angiocrine factors which support correct specification and differentiation of neural cell types, ensuring regionalization along the dorsoventral and anteroposterior axes [38–40].

Not all neurogenic niches are regulated by oxygen levels, however. In the developing hindbrain, for example, the vasculature regulates neurogenesis independently of oxygen levels [41]. Thus, both oxygen levels and vascular-derived factors appear necessary to regulate neurogenesis [42].

While hypoxia and its downstream effects serve as the first indication of a vascular contribution to embryonic neurogenesis, vascularization has been shown to regulate neural development in several ways. For example, blood vessels are able to regulate neurogenesis through the secretion of angiocrines such as VEGF-A [23,38,43]. While VEGF-A is secreted by neural cells to induce vascularization, reciprocally, ECs secrete this factor to influence neural development. Haigh *et al.* [44] showed that deletion of VEGF-A resulted in telencephalic devascularization and hypoxia which subsequently caused decreased neuronal proliferation in the ventricular and sub-ventricular zones [44]. Additional findings have reported that mouse mutants lacking VEGF-A expression in ECs also incur cortical malformations [33,45].

As a final example of the importance of vasculature, its integration with the developing CNS may also aid in neural cell migration. The secretion of inhibitory neurotransmitters and stromal-cell-derived factor-1 (SDF-1) by ECs enables migration of GABAergic neurons and Cajal–Retzius cells, respectively [46–48]. Vasculature also serves to guide the path of neuroblasts as well as integrate mature neurons into neural circuits [33,49].

## 3. Enigmatic roles of endothelial-derived factors in neural tube development

### 3.1. The role of vasculature in neural tube development

While it is evident that vasculature plays an important role in CNS development, focusing on studies investigating the role vasculature might play in NTDs offers the unique benefit of specifying its importance in NT development rather than general CNS development.

In 1987, Stevenson *et al.* proposed the hypothesis that abnormal blood vessel supply early in neural development is causative in NTDs [50]. This study focused on the relationship between NTDs and arterial abnormalities in six fetuses and found that these fetuses demonstrated severely abnormal arterial supply to the region of the CNS defect. The authors rationalized that since angiogenesis precedes neurogenesis in embryogenesis and that these arteries would thus have developed prior to the completion of NTC, the correlation seen between blood vessel aberrations and NTDs could be presumed to be the cause of blood vessel aberrations causing NTDs, rather than the other way around. This led the authors to theorize that abnormal vasculature leading to inadequate nutrient supply to the rapidly growing neural folds results in NTDs.

This hypothesis presents an immensely valuable tool for investigating the role of vasculature in early CNS development. Considering many mechanisms involved in NT development remain elusive, utilizing NTDs, which are easily identifiable as an incompletely or abnormally closed NT [51], as a clear indication of disrupted NT development, is useful when studying the roles various factors might play in neurulation.

Further supporting this hypothesis, a 2007 study noted NTDs in mutant mouse embryos with inactivated endothelial Survivin [52]. Survivin, an apoptosis inhibitor, serves as a mitotic regulator and is thus highly expressed in rapidly proliferating ECs. These mutant embryos, in addition to presenting significant vascular abnormalities, also presented with NTDs of varying severity (30% of mutant embryos) at E9.5, E10.5 and E12.5. While NTDs do commonly occur in lethal embryonic phenotypes, this is typically noted in cases when developmental retardation occurs prior to or during NTC itself, whereas, in this case, developmental retardation occurred only at E12.5, after the latest presentation of NTDs. Additionally, it was noted that these NTDs were not caused by bleeding resulting from the vascular aberrations. As a result, it was suggested that these defects were likely the result of disruptions to the release of endothelium-derived soluble factors, which are otherwise involved in neural proliferation and differentiation.

### 3.2. Vasculogenesis prior to neurulation

When discussing the role of the endothelium on NT development, most papers address the vascularization of the NT from the formation of the PNVP onwards, but rarely mentioned is the role of the endothelium prior to this period.

Considering that vascularization of the NT only occurs after its closure, in order to thoroughly evaluate the role of blood vessels in NT development, investigation of the potential endothelial-derived factors present during this earlier time period is essential. Thus, while vascularization of the NT itself follows NTC, vascularization events commencing prior to the onset of neurulation might play a significant role in the occurrence of NTDs, indicating its necessity in neurulation. There is considerable evidence suggesting that vascularization of the yolk sac preceding the formation of the NT might be necessary for efficient NT formation and morphogenesis.

It has also been theorized that insults to vasculogenesis occurring before NTC may cause NTDs [53]. By extension, the observation of NTDs in embryos with interruptions to this early vasculogenesis would suggest a requirement for endothelial-derived factors on NT formation prior to NTC.

The yolk sac is the first site of both erythropoiesis and vasculogenesis in mice and humans [54], with primitive blood cell formation beginning from day 16 to day 19 in humans [54,55] and roughly E6.5 in mice [56]. Before the establishment of blood vessels through the placenta (hemotrophic nutrition), nutrients are obtained by histotrophic mechanisms, including endocytosis by phagocytosis, pinocytosis, and receptor-mediated endocytosis from the maternal environment [6].

Indeed, the importance of the endocytic apparatus in the delivery of nutrients to the neurulating embryo is evident in the incidence of NTDs and early embryonic lethality observed in mouse knockout studies, which have successfully disrupted components of the pathway [57–61]. Moreover, there is a growing body of evidence suggesting the yolk sac vasculature plays a significant role in NT formation. This research focuses on the role of maternal diabetes-induced NTDs.

It has been demonstrated that pregestational maternal diabetes mellitus contributes to NTDs; however, it also contributes significantly to other congenital malformations, most notably those of the cardiovascular system [62,63]. Early manifestations of this cardiovascular dysregulation involve yolk sac vasculopathy and disrupted yolk sac capillaries [64]. This resulting yolk sac vasculopathy is speculated to underly the formation of the NTDs seen in gestational diabetes [63,65,66]. To investigate signalling disruptions in gestational diabetes, Cao *et al.* [53] utilized a transgenic mouse model expressing BMP4 in order to restore diabetes-inhibited BMP4 expression in Flk-1+endothelial progenitors and prevent diabetes-induced vasculopathy. Loss of BMP4 expression was shown to be associated with vasculopathy as well as NTDs, leading the authors to initially hypothesize that diabetes-induced interruptions of BMP4 signalling might be a common causative factor in both the resulting vasculopathy and NTDs. The results revealed that BMP4 transgenic expression reduced the incidence of NTDs from 24.14% to 6.45%, supporting the hypothesis that BMP4 transgenic expression during vasculogenesis was able to prevent maternal diabetes-inducing NTDs. However, BMP4 transgenic expression took place during vasculogenesis; additionally, it was noted that BMP4 transgenic expression was successfully able to prevent maternal diabetes-induced vasculopathy. Since it has been well established that embryonic vasculopathy is linked to NTD formation [67–69], the amelioration of NTDs in these transgenic mice may very well be the result of reduced vasculopathy, in addition to a potential direct role of BMP4 on neurulation.

This study further indicates a potential link between EC activity and NT formation. It was also suggested that correction of the disrupted vascular signalling in maternal diabetes may be sufficient to prevent diabetic embryopathy, including NTDs.

## 4. Organoid models

### 4.1. Recent advancements in neural tube organoid models

As it has already been established by others that endothelial-derived factors support NT development, the question raised in this review is whether the inclusion of these factors is necessary to achieve accurate recapitulation of NT development *in vitro*.

Within conventional cell culture systems, oxygen delivery to the cells, via diffusion through the medium, is generally sufficient to support cell survival. However, the culture of 3D models, as well as tissue-specific oxygen consumption rates, complicate this issue and raise significant queries about the efficacy of current cell culture conditions.

Firstly, the oxygen-sensitive enzyme pathway associated with HIF is controlled by a specific setpoint of oxygenation which varies with tissue-specific oxygen consumption rates [70,71]. Therefore, the oxygenation levels required for correct activation of these pathways, such as for the purposes of NPC differentiation in developing neural tissue, will vary between different cell types being cultured. For this reason, within the context of this system, the term ‘hypoxia’ is somewhat redundant; that is, there is no one specific oxygen concentration that confers hypoxic conditions for all cell types.

Additionally, in 3D cultures, cells further from the aggregate surface experience lower oxygen concentration due to hindered diffusion. *in vivo*, the maximum distance between cells and capillaries rarely exceeds 200 µm; in fact, they are typically found less than 100 µm away from one another [72]. As these aggregates, or organoids in the case of NTOs, reach a size of around 200−400 µm [73], neural organoids specifically have been shown to develop hypoxic cores. These hypoxic cores often become necrotic, hampering the growth and development of the tissue significantly and often acting as a limiting factor in organoid culture periods and developmental complexity [74].

In terms of nutrient provision, factors necessary for cell survival and growth are largely provided for by careful calibration of cell culture media. Basal media such as DMEM/F-12 [75–77] and mTeSRT-1 [78,79] include essential nutrients such as glucose, amino acids, vitamins and essential salts and are often further supplemented as needed, accounting for most metabolic and energy demands of dividing and differentiating cells.

However, these media do not necessarily account for endothelial-derived factors necessary for neural development. If we knew what these factors were, such as in the case of BMP4 signalling [53], there is a possibility that we could supplement medium with factors currently missing and that this supplementation might account for differences between *in vivo* phenomena and *in vitro* models, which arise due to insufficient pathway activation.

Thus, while a majority of factors are accounted for in current culture protocols; even so the emerging evidence presenting various roles of endothelial-derived factors in NT formation emphasizes the need to better characterize these factors if they are to be incorporated into NTO generation, to more accurately recapitulate NT development. The prominent shortcoming in improving cell culture conditions is, thus, our limited understanding of the endothelial-derived factors influencing neural development and the mechanisms by which they contribute to this process.

### 4.2. Vascularized and avascular neural organoids

Under the assumption that vascularization is necessary for accurate recapitulation of NT formation, how might we expect these NTOs to differ from current avascular organoids? Considering that currently a vascular NTO model has yet to be generated, comparing vascular and avascular NTOs is not directly possible; however, we can look at available vascularized neural organoids and note the advantages vascularization has afforded these models.

Avascular cerebral organoids (COs) have been surmized to demonstrate limited developmental complexity, corresponding to immature fetal states, exhibiting fewer distinct cell types, and inconsistent electrophysiological activity [80–82]. On the other hand, attempts to vascularize neural organoids have presented encouraging results, demonstrating the mitigation of a number of these limitations.

The most anticipated advantage observed in vascularized neural organoids is reduced cell death owing to enhanced oxygen and nutrient delivery. This integration of vasculature enables better penetration of oxygen into the centre of the organoids, preventing the development of hypoxia and necrosis. Additionally, the formation of functional vasculature and neurovascular units enables interactions between neural and ECs. One major effect of these interactions is reduced apoptosis, supporting the development and functionality of these organoids [12,83]. Organized vascular structures have also demonstrated upregulation of Wnt/β-catenin signalling [84]. Similarly, the formation of vascularized brain organoids (BOs) resulted in complex tubular vessels and networks specific to those in the human cerebrum. These have been noted to effectively recapitulate early vasculogenesis and angiogenesis and, most valuably, vascularization of the NT by formation of a PNVP and endothelial sprout invasion [85]. Importantly, BOs have shown considerable increases in neural progenitor pools alongside reduced apoptosis [12]. This reduction in apoptosis has resulted in a significant reduction in hypoxia, specifically of the interior tissues, which have been shown to become necrotic in avascular organoids [83].

This alleviated hypoxia, in turn, enables longer culture periods and appears to result in neuronal cells of higher maturity. One study demonstrated that vascularized cortical organoid-derived neurons resembled *in vivo* neurons of gestational weeks 16−19, compared to avascular controls which resembled an earlier gestational stage, roughly between weeks 0 and 12 [83]. Additionally, a reliable indicator of neuronal maturity is spontaneous electrical activity. Vascularized organoids demonstrated spontaneous electrical activity, indicating the presence of both chemical and electrical synapses [83,86]. Vascularization of organoids has also successfully encouraged the incorporation of microglia able to respond to immune stimulation [12,85].

It has also been noted that the accurate recapitulation of the blood–brain barrier in vascularized BOs is promising for future disease and drug testing [12,83]. In a similar way, vascularized NTOs may provide a useful and accurate model to study early neurovascular defects in NT formation, as well as a method for testing potential pharmaceutical interventions.

Overall, studies vascularizing neural organoids have been able to demonstrate that without vasculature, BOs are limited in their differentiation capabilities, cell diversity and maturity, and electrophysiological activity. On the other hand, studies that have co-cultured ECs with COs have noted accelerated neural differentiation, decreased apoptosis and improved electrical activity when compared to avascular counterparts [83,84,86,87]. Based on the vascular requirements for tissue-specific oxygenation and endothelial-derived factors in neural organoids, it follows that vascularization would considerably improve the development of complex and functional NTOs.

### 4.3. Vascularization of neural organoids

There are a number of methods that have been utilized to successfully vascularize neural organoids. When looking to pioneer the vascularization of NTOs in the near future, researchers might use existing strategies and adapt them as needed.

**Figure 2.**
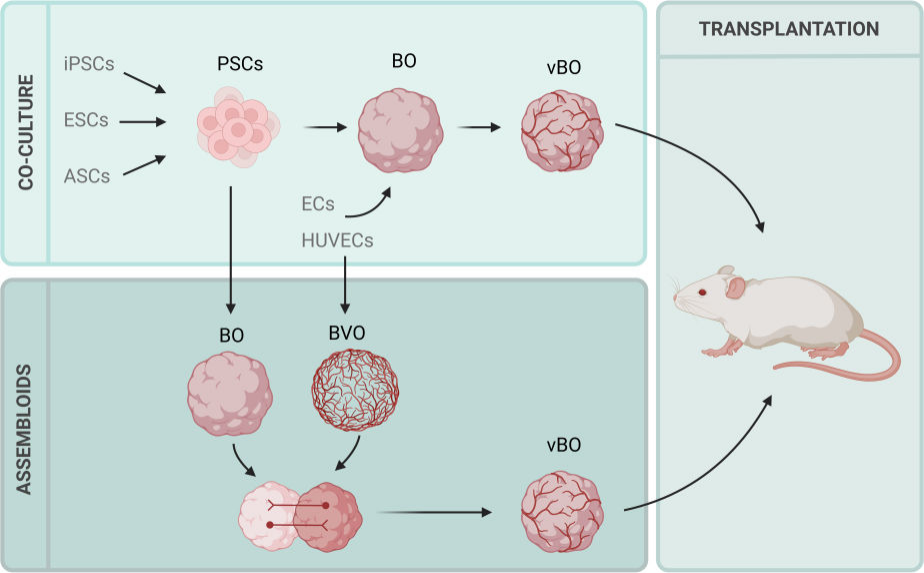
Common methods of neural organoid vascularization. Methods of vascularizing neural organoids have largely employed co-culture of neural organoids with ECs or HUVECs in order to develop vBOs. Another method is the formation of assembloids; this approach involves the formation of separate BOs and BVOs, following which these organoids are merged to form a vBO. To enable circulation within vBOs, co-cultured or assembloid vBOs are often transplanted into hosts such as mouse brains. Image created in BioRender [88].

One of the simplest methods of vascularizing neural organoids is co-culture with endothelial or vascular cells. This involves the addition of ECs with neural organoids, enabling ECs to integrate alongside neural development. In this method, ECs are cultured with developing BOs, enabling the simultaneous development and integration of these two systems. Human umbilical vein endothelial cells (HUVECs) are also commonly utilized due to their wide accessibility. This method is outlined in figure 2.

As an example, Ahn *et al.* [89]initially generated and separately cultured human COs and human blood vessel organoids (BVOs), and then separated the vascular plexuses from the BVOs and co-cultured these with COs, before neural cell differentiation is expected to take place. The findings from this study showed that vascular cells arising from these BVOs were able to penetrate the COs and went on to form neural-specific blood vessel networks that were able to be maintained for over 60 days [89]. Pham *et al.* additionally co-cultured BOs and vascular-derived vascular cells, which resulted in penetrating vessels expressing the endothelial marker platelet EC adhesion molecule [90]. Shi *et al.* [86] used a similar approach, co-culturing human BOs with HUVECs. This study demonstrated that compared to non-vascularized BOs (vBOs), those co-cultured with HUVECs grew larger and significantly decreased hypoxia, indicated by decreased HIF1-positive cells and cleaved caspase3-positive apoptotic cells, altogether indicating a positive effect on NPC proliferation and/or neuronal survival. Encouragingly, they found accelerated neurogenesis and early neural development in these BOs in addition to better growth and functional development, while electrophysiological analysis revealed that higher spontaneous active neuron activity was present in these vascularized BOs and that this facilitated neuronal maturation.

Another method of vascularization includes the formation of assembloids, the fusion of organoids of different tissue types [12]. In this case, separate neural organoids and vascular organoids are cultured and these organoids are then fused to form a neurovascular assembloid. This technique was employed by Sun *et al.* who cultured separate BVOs and BOs and upon fusion, formed vBOs [12].

While these techniques have proven effective in advancing the development of neural organoids, one remaining issue is the lack of circulation through the resulting integrated blood vessels [87]. For this reason, *in vivo* transplantation into host organisms has become an attractive addition to vascularization protocols [83,86,90,91]. In these methods, pre-vascularized neural organoids are implanted into a host organism, most commonly immunodeficient mice, allowing the host vasculature to integrate with the existing vasculature of the implanted organoid, enabling a more accurate representation of *in vivo* circulation. Most commonly, organoids are transplanted into mouse brains [86,90,91]; however, they have also been successfully transplanted into subcutaneous tissue of the hind limbs [83]. These transplantation models have successfully induced functional circulation of transplanted organoids compared to avascular counterparts [83,86,91].

Compared to age-matched, avascular counterparts, the transplanted vBOs showed reduced apoptosis, were able to grow larger in size and contained a significantly larger number of NeuN-positive mature neurons, indicating improved neural maturity in these transplanted vascularized organoids [91]. Highlighting the importance of this functional vascularization, while avascular counterparts were unable to survive, transplanted vascularized organoids survived for 233 days post-transplantation. This increased lifespan is thought to be due to the vital support of oxygen and nutrient delivery [91].

As previously described, Shi *et al.* pre-vascularized their BOs by co-culturing them with HUVECs *in vitro*, but they then transplanted their pre-vascularized BOs and compared these to transplanted organoids which were not pre-vascularized. Findings from this study showed that pre-vascularization resulted in enhanced blood vessel formation and reduced apoptosis in the transplanted organoids compared to the non-pre-vascularized organoids [86]. Additionally, Cakir *et al.* noted enhanced organoid survival in transplanted organoids which were able to survive for 30 days post-transplantation compared to non-pre-vascularized controls which became undetectable 10−30 days post-transplantation [83]. Immunohistochemical analysis also revealed that both HUVEC-derived and mouse-derived ECs were present in the capillaries of the transplanted BOs, suggesting successful integration of the transplanted and host vessels [86]. Moreover, only the pre-vascularized organoids demonstrated successful infiltration of host blood vessels and the establishment of functional perfusion [83]. These results show not only the benefits of circulation in neural organoids but also emphasize the importance of methodology, suggesting that both pre-vascularization and the establishment of functional perfusion via circulation are necessary for organoid development and survival, at least within these human BOs.

These rapidly advancing methods of vascularization are not without limitations, however. Firstly, the co-culture of ECs with BOs involves unanswered questions regarding the timing of co-culture, the ratio of ECs found in each organoid, and the time points at which various steps in the process should take place to accurately recapitulate *in vivo* development [92]. Co-culture with ECs alone is also insufficient to recreate the full vascular structure and still requires exogenous VEGF to induce angiogenesis. The crosstalk between stem cells and ECs is also unclear, complicating the direct addition of stem cells and ECs and leading to unpredictable differentiation [93]. The generation of circulation is currently not present in *in vitro* vascularized organoids due to lacking a pumping system. Transplantations into host organisms have provided a useful solution to this issue but still present additional obstacles. Firstly, due to the genome variance between human organoids and the host organisms, it’s uncertain whether vascular interactions will vary as a result of species-specific differences.

Another mode of producing convective blood flow is the combination of microfluidic devices with these organoid cultures; however, while these have proved most efficient in terms of reducing hypoxia, they have not addressed other important aspects of neurovascular maturity [94]. For this reason, biological self-organized vessels, such as those in transplanted organoids, are likely the best suited to recapitulate neurovascular development, though their own limitations should be carefully considered.

## 5. Conclusions

While NTOs have presented an exciting and valuable tool in embryology, our inability to compare these models to their *in vivo* counterparts makes evaluating their accuracy challenging. As these models become more complex, this poses a potential issue when making inferences about *in vivo* development.

The suggested reliance on endothelial-derived factors for CNS development emphasizes a potential limitation in current avascular NTO models. While nutrient demands are largely met with carefully calibrated cell culture medium and supplementation, the open question regarding what unknown endothelial-derived factors might affect neurulation means factors necessary for accurate development might not be available to these organoids, and thus, this might affect their reliability.

Vascularization of neural organoids has shown promising results, suggesting that vascularized NTOs may show higher levels of complexity, longer culture periods, and higher maturity than current avascular models. Although it is fair to say that, based on research findings, vascularization of NTOs is likely necessary to improve their complexity and accuracy, further advancements in NTOs hinge on our ability to clearly define the roles of endothelial-derived factors in developmental processes. It is still unclear whether findings from future research will reveal that endothelial-derived factors can be supplemented by manipulating cell culture conditions; however, until this issue can be addressed, it appears that working towards a functionally vascularized NTO holds the most promise for research applications. The improvements vasculature might afford these models will contribute to not only our understanding of this critical period of embryological development but can also be used as more accurate disease models to study the neurovascular contributions to NTDs, as well as a potential drug model.

## Data Availability

This article has no additional data.
